# Retention Rate of Ixekizumab in Psoriatic Arthritis: A Real-World Study

**DOI:** 10.3390/jpm14070716

**Published:** 2024-07-03

**Authors:** Elisa Bellis, Piero Ruscitti, Denise Donzella, Gloria Crepaldi, Valeria Data, Marinella Gammino, Mariele Gatto, Valeria Guardo, Claudia Lomater, Elena Marucco, Marta Saracco, Annamaria Iagnocco

**Affiliations:** 1 Academic Rheumatology Centre, Department of Clinical and Biological Sciences, University of Turin, AO Mauriziano di Torino, 10128 Turin, Italygcrepaldi@mauriziano.it (G.C.);; 2 Rheumatology Unit, Department of Biotechnological and Applied Clinical Sciences, University of L’Aquila, 67100 L’Aquila, Italy

**Keywords:** drug retention rate, ixekizumab, psoriatic arthritis

## Abstract

We aimed to examine the drug retention rate (DRR) of the interleukin-17 inhibitor ixekizumab in a real-world monocentric cohort of psoriatic arthritis (PsA) patients and to assess the predictors of drug discontinuation. Consecutive PsA patients who underwent treatment with ixekizumab from October 2019 to February 2023 were enrolled in this observational, retrospective, monocentric study. Clinical records were assessed at baseline and throughout the follow-up period. We collected sociodemographic data, smoking habits, body mass index, the presence of Human Leukocyte Antigen B27, comorbidities, disease involvement and duration, previous therapy, discontinuation of ixekizumab, reasons for discontinuation, and adverse events (AEs). DRR was evaluated as time to drug discontinuation and assessed through Kaplan–Meier curves. Baseline factors predicting drug discontinuation were investigated through logistic regression models. Eighty PsA patients were included in this study. Ixekizumab was administered at a dose of 160 mg by subcutaneous injection at baseline, followed by 80 mg every four weeks thereafter. Ixekizumab had a 38-month-cumulative DRR of 43.8%, accounting for both inefficacy and AEs. When considering only inefficacy, the DRR was 62.6%. Comorbidities (*p* = 0.665), obesity (*p* = 0.665), smoking (*p* = 0.884), disease duration ≤ 2 years (*p* = 0.071), axial (*p* = 0.131) and skin involvement (*p* = 0.460), and previous therapies, including conventional synthetic (*p* = 0.504) and biological (*p* = 0.474) Disease-Modifying Antirheumatic Drugs (bDMARDs), as well as the number of previous bDMARDs or targeted synthetic Disease-Modifying Antirheumatic Drugs (tsDMARDs), did not significantly affect the DRR (*p* = 0.349). Multivariate analysis found no independent predictors of drug discontinuation. The most frequent AEs leading to discontinuation were skin reactions; no severe infections were observed. In our real-world study, comorbidities, disease duration, and previous therapies did not affect the DRR of ixekizumab. Ixekizumab had a favorable safety profile, with no severe AEs observed.

## 1. Introduction

Psoriatic arthritis (PsA) is complex disease characterized by a combination of musculoskeletal manifestations, such as peripheral and axial involvement, dactylitis, and enthesitis, along with various extra-articular abnormalities [1]. PsA affects 24% of individuals with psoriasis (PsO) [1], yet it can go undiagnosed in up to 30% of these cases [2]. Subclinical enthesitis is detectable in up to 36% of PsO patients [3], while synovitis is detected in 50.7–85.0% of these patients across various studies [4,5,6]. Although PsO typically precedes the onset of arthritis, 15% of patients exhibit both PsO and joint symptoms simultaneously, and 20% develop PsA before skin involvement [1]. The diverse clinical manifestations of PsA make its diagnosis particularly challenging. Early diagnosis and treatment, however, are fundamental to prevent joint damage, as they are linked to improved long-term outcomes [1] and better patient-reported outcomes [7]. Early treatment in a prodromal phase in patients with PsO experiencing arthralgia could also help prevent the onset of PsA [8].

PsA patients, moreover, often present with several comorbidities that need careful consideration during the management of the disease, such as cardiovascular disease and metabolic disorders [9].

Interleukin 17 (IL-17) plays a key role in the pathogenesis of PsA, contributing to inflammation, especially in the skin and entheses. Specifically, IL-17A, through its interaction with its receptor, triggers proinflammatory effects in neutrophils and macrophages. These proinflammatory effects also involve keratinocytes and endothelial cells. At the level of endothelial cells, IL-17A may increase procoagulant activity, thus contributing to endothelial dysfunction and increasing the risk of cardiovascular comorbidities [10,11]. Ixekizumab, a monoclonal antibody directed against IL-17A, prevents its binding to the IL-17 receptor [12], and it is approved by the European Medicines Agency for the treatment of PsA, plaque PsO, and axial spondyloarthritis [13].

The early use of IL-17 inhibitors in PsO patients at high risk for PsA has been shown to reduce joint pain and subclinical inflammation, suggesting potential for PsA interception strategies [8].

In patients diagnosed with PsA, however, the phase 3 randomized controlled SPIRIT-P1 and SPIRIT-P3 trials showed that ixekizumab effectively reduces disease activity and radiographic progression, as well as in improving patients’ function and quality of life in biologic-naïve individuals [14]. In the SPIRIT-H2H, Ixekizumab demonstrated greater efficacy than adalimumab in achieving concurrent improvements of joint and skin involvement among biologic-naïve PsA patients [15,16]. Additionally, in the SPIRIT-P2 study, ixekizumab improved signs, symptoms, and patient-reported outcomes, even in patients previously treated with tumor necrosis factor inhibitors (TNF-i) [17,18,19], maintaining a favorable safety profile [14,17,20,21].

There are increasing amounts of data regarding the real-world efficacy and safety of patients with PsO who are treated with ixekizumab [22,23,24,25,26,27,28,29,30,31,32,33,34,35,36,37,38,39], and currently, interest in real-world data regarding its efficacy and safety profile in PsA is increasing [31,40,41,42,43,44,45,46,47,48,49,50,51,52]. Indeed, real-world studies offer additional clinical insights into the disease in a heterogenous context such as the clinical setting, when patients comorbidities and contextual factors can impact the management of the disease and its outcome as well as the drug retention rate (DRR) [53,54].

The objective of this study is to examine the DRR of ixekizumab in a real-world monocentric cohort of PsA patients and to assess the predictors of drug discontinuation. 

## 2. Materials and Methods

This is an observational, retrospective, non-profit study, involving consecutive adult patients (≥18 years) affected by PsA, and follow-ups were performed at the Academic Rheumatology Centre of Clinical and Biological Sciences Department of Turin University—Turin, Mauriziano Hospital, Italy, in patients who underwent treatment with ixekizumab from October 2019 to February 2023. 

Inclusion criteria encompassed (i) adult age; (ii) fulfilment of CASPAR classification criteria for PsA [55]; and (iii) availability of complete data records. Exclusion criteria were: (i) less than two follow-up visits and (ii) inability to provide informed consent.

Clinical and laboratory data were extracted from patients’ medical records. Specifically, we collected the following information: sociodemographic data, smoking habits, body mass index, the presence of Human Leukocyte Antigen B27 (HLA-B27), comorbidities, phenotypes of PsA, disease duration, previous and concomitant therapies, occurrence of adverse events (AEs) and infections, any withdrawal of ixekizumab, and the cause for discontinuation.

Considering the retrospective study design, no specific sample size was estimated for this study.

The local Institutional Ethics Boards approved the study (Interagency Territorial Ethics Committee A.O.U. Città della Salute e della Scienza, No. 139/2023), and informed consent was obtained from all participating patients.

### Statistics

Firstly, descriptive statistics were provided. Dichotomic variables were expressed as percentage whereas continuous variables as mean ± standard deviation (SD) or median and range interquartile range (IQR) according to their distribution. Kaplan–Meier curves were built to evaluate the cumulative DRR of ixekizumab with the event being drug discontinuation due to inefficacy and/or AEs. Furthermore, Kaplan–Meier curves were performed to evaluate the influence of patient clinical characteristics on the DRR of ixekizumab. Survival curves were thus compared by using the log-rank test. To exploratively assess possible baseline predictors of drug discontinuation, age- and male sex-adjusted multivariate regression logistic models were exploited. The purposeful selection process of covariates started by a univariate analysis of each variable and their clinical relevance. At the end of this multistep process of deleting and refitting, age- and sex-adjusted multivariate models were built, providing odds ratio (OR) estimations of significant associations between clinical features and drug discontinuation. Disease duration ≤ 2 years (lacking specific definition of early PsA [56]), HLA-B27, the presence of comorbidities and obesity, axial involvement and concomitant active skin disease, previous therapies with either conventional synthetic Disease-Modifying Antirheumatic Drugs (csDMARDs) or biological Disease-Modifying Antirheumatic Drugs (bDMARDs), were selected as possible characteristics impacting the drug discontinuation considering univariate analyses but also their clinical relevance according to available literature [57]. Different age- and male sex-adjusted multivariate regression logistic models were also built, taking into account the number of patients discontinuing the drug in this cohort. 

Correlation analyses were also estimated among the cumulative number of previous bDMARDs/targeted synthetic Disease-Modifying Antirheumatic Drugs (tsDMARDs) and clinical features. 

The statistical significance was set at *p* < 0.05 and all *p*-values were two-sided. The Statistics Package for Social Sciences (SPSS for Windows, version 22.0, SPSS Inc., Chicago, IL, USA) was used to exploit both regression and correlation analyses, and GraphPad for Windows (version 8.0, San Diego, CA, USA) was used for the assessment of DRR.

## 3. Results

Eighty patients with PsA were included in the study and followed up on for a median of 12 (IQR 23.2) months. The baseline clinical features of the whole patient cohort are summarized in Table 1. 

At the first observation, the mean age was 50.1 ± 11.8 years. Twenty-four (30.0%) of the patients were male, twenty-seven (33.8%) were obese, and twenty-two (27.5%) were smokers.

Forty-six (57.5%) patients presented comorbidities, and the main comorbidities were high blood pressure (n. 36, 45.0%), type 2 diabetes (n. 17, 21.3%), fatty liver disease (n. 17, 21.3%), cardiovascular disease (n. 16, 20.0%), dyslipidemia (n. 12, 15.0%), and kidney disease (n. 5, 6.3%).

The median disease duration was 4 (IQR 9) years. All patients presented peripheral joint involvement; in addition, 25 (31.3%) displayed axial and 35 (43.8%) enthesis involvement. Sixty-eight (85%) showed skin involvement, and nineteen (23.8%) psoriatic onychopathy. 

All patients received ixekizumab at a dose of 160 mg by subcutaneous injection at baseline, followed by 80 mg every four weeks thereafter.

Sixty-five (81.3%) patients had received csDMARDs before ixekizumab, with the majority (n. 61, 76.3%) receiving methotrexate. Forty-six (57.5%) patients had received at least one TNFi, four (5%) received one interleukin 12/23 inhibitor (IL-12/23i), nine (11.3%) received a tsDMARDs, and twelve (15%) had already been treated with an IL-17i medication. Among patients previously treated with secukinumab, five (41.7%) discontinued ixekizumab during follow-up, of whom three (25%) ended the treatment because of ineffectiveness. Fifty-six (70.1%) had experienced a failure with at least one bDMARD/tsDMARD and nine (11.3%) to three or more bDMARDs/tsDMARDs before ixekizumab.

Forty-three (53.8%) patients were on ixekizumab at the last follow-up visit. Among those who discontinued the treatment, 25 (31.3%) did so due to inefficacy, with 9 (11.3%) experiencing primary inefficacy and 10 (12.5%) experiencing secondary inefficacy, while 12 (15%) discontinued treatment due to AEs (Table 1). 

### 3.1. Drug Retention Rate 

**Figure 1:**
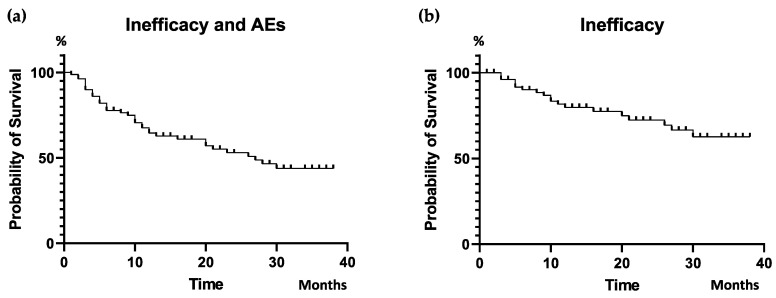
Cumulative DRR of ixekizumab: Inefficacy and AEs (**a**) and Inefficacy (**b**). DRR, drug retention rate; AEs, adverse events.

Ixekizumab was administered in our cohort for a median of 12 (IQR 23.2) months. Taking follow-up into account, the cumulative 38 month DRR of ixekizumab was estimated to be 43.8%, considering the discontinuation for both inefficacy and side effects, whereas in 62.6%, the interruption was only due to inefficacy (Figure 1). 

**Figure 2:**
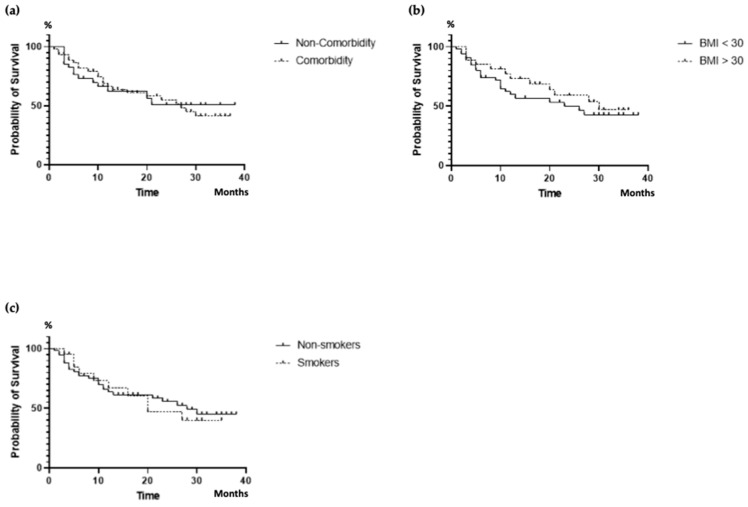
DRR of ixekizumab according to comorbidities (**a**), obesity (**b**), and smoking (**c**). DRR, drug retention rate; BMI, body mass index.

After stratifying the results, it was evident that the DRR of ixekizumab was unaffected by concomitant comorbidities (*p* = 0.993), obesity (*p* = 0.665), and smoking habit (*p* = 0.884) (Figure 2). 

**Figure 3:**
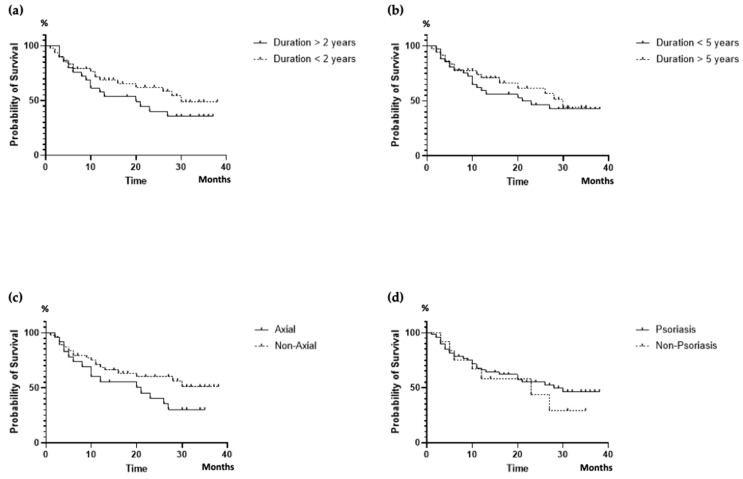
DRR of ixekizumab according to disease duration (**a**,**b**), axial disease (**c**), and skin involvement (**d**). DRR, drug retention rate.

Despite a positive trend noted in patients with a disease duration ≤ 2 years (*p* = 0.071), the DRR did not appear to be significantly influenced by the disease duration. DRR was not significantly different (*p* = 0.062) between patients with axial and peripheral involvement (*p* = 0.131), despite a numerically higher proportion of patients with peripheral involvement persisted on treatment in the long term. Skin involvement did not influence the DRR of ixekizumab (*p* = 0.460) (Figure 3).

**Figure 4:**
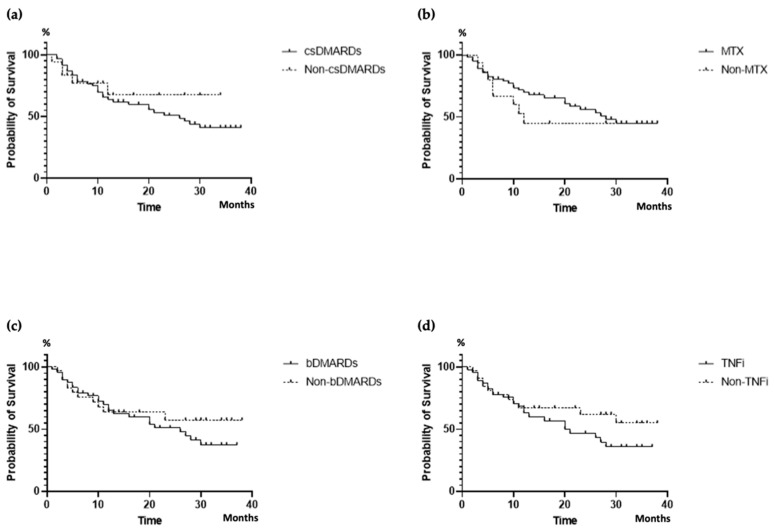
DRR of ixekizumab according to previous therapies: csDMARDs (**a**), MTX (**b**), bDMARDs (**c**), TNFi (**d**). csDMARDs, conventional synthetic Disease-Modifying Antirheumatic Drugs; MTX, methotrexate; bDMARDs, biologic Disease-Modifying Antirheumatic Drugs; TNFi, tumor necrosis factor inhibitor; DRR, drug retention rate.

In addition, the DRR did not seem to be influenced by previous therapies, including csDMARDs (*p* = 0.504), methotrexate (*p* = 0.396), bDMARDs (*p* = 0.474), and previous TNFi (*p* = 0.247) (Figure 4). The number of previous bDMARDs/tsDMARDs was found to be correlated with patients’ age (r = 0.310, *p* = 0.005) and disease duration (r = 0.265, *p* value 0.018), while it did not appear to affect the duration (r = 0.106, *p* = 0.349) or discontinuation (r = 0.042, *p* = 0.896) of ixekizumab therapy.

When conducting multivariate explorative analyses to evaluate the possible predictive role of selected baseline clinical variables in the likelihood of ixekizumab discontinuation, none of these variables reached statistical significance (Table 2). Disease duration and the presence of HLA-B27 were not found to be predictive of ixekizumab discontinuation. Similarly, patient clinical characteristics (i.e., comorbidities, obesity, axial disease, and skin involvement) did not appear to influence drug discontinuation. In addition, the prior therapies with csDMARDs and bDMARDs were not identified as predictors of ixekizumab discontinuation.

### 3.2. Adverse Events

The most frequent AEs leading to the discontinuation of ixekizumab (Table S1) were skin reactions, including localized reactions at the injection site (four cases, 5.0%) or diffuse skin reactions (three cases, 3.75%). Three (3.75%) patients discontinued the treatment due to diarrhea, and one of these was thereafter diagnosed with microscopic colitis. This patient had previously presented with diarrhea and gastrointestinal intolerance to csDMARDs. Another patient received a concomitant diagnosis of lung malignancy just after the initiation of therapy, despite prior screening. Only one patient had to discontinue ixekizumab due to an infection (i.e., persistent oral candidiasis). Eight (10%) patients experienced SARS-CoV-2 infection, which was not severe in all cases. A single case of localized Herpes Zoster and 1 pyelonephritis was reported.

## 4. Discussion

The majority of real-world data in the literature focus on patients with PsO, while fewer studies concentrate on those affected by PsA [58].

In this study, we showed that ixekizumab entails a good DRR in a real-life PsA cohort, with a good safety profile. 

Taking into account the complete follow-up period of our cohort, a 38-month cumulative DRR was seen in 43.8%, where discontinuation was due to both inefficacy and AEs, whereas in 62.6%, interruption was due to inefficacy only. These findings are consistent with previous real-life data in PsA patients treated with ixekizumab. Recently, Braña et al. observed a 12-month DRR of 65% in a retrospective monocentric cohort of 72 patients [44]; similarly, an analysis of administrative claims databases from the USA highlighted that 52.8% of patients maintained ixekizumab therapy for a 12 months follow-up period [59]. Glintborg et al., drawing from data across five Nordic biologics registries, outlined a 12-month DRR of 57–65% for 361 treatment courses [45]. Joven et al. instead highlighted a drug persistence rate of 68.5% at two years [43]. In a monocenter cohort study conducted in the UK involving spondyloarthritis patients undergoing therapy with the IL-17i ixekizumab and secukinumab, drug survival rates were found to be 69% at 12 months and 60% at 24 months [49]. Takami et al. reported a 10-year DRR of 50% for ixekizumab in a long-term follow-up study [50].

In the literature, the DRR of ixekizumab has been found to be comparable to that of other biologics, with the exception of a higher DRR compared to infliximab [42].

Our PsA patient cohort exhibited a high rate of obesity and several comorbidities, primarily cardio-metabolic, which aligns with previous research findings [40,43,60]. Nevertheless, when analyzing factors that may influence the DRR of ixekizumab, it became evident that obesity had no significant effect, as previously noted in the literature [43,44]. Similarly, in our study, the presence of comorbidities and smoking habits, as previously documented by Braña et al., did not show any significant influence on the DRR of ixekizumab [44].

All our patients presented with peripheral involvement. Interestingly, axial involvement appeared to influence the DRR with a negative, albeit non-significant, trend, whereas this was not observed when considering skin involvement. It is necessary, nevertheless, to confirm this trend on larger cohorts, as the evaluation of these factors is crucial when tailoring PsA patients’ treatment, given their different phenotypes and multiple comorbidities [60].

Moreover, we highlighted a positive, but not statistically significant, trend for a higher DRR in patients with a disease duration shorter than 2 years. Braña et al. noted, however, that disease duration did not influence drug persistence [44].

The majority of our patients (70.1%) had encountered prior treatment failure with at least one bDMARD/tsDMARD, and 30.1% had experienced multiple treatment failures. Moreover, 15% had already been treated with an IL-17i. However, prior treatments with csDMARDs, including methotrexate, bDMARD/tsDMARD, or even a number of previous bDMARD/tsDMARD treatments, did not exert a significant impact on the DRR. Similarly, in the PRO-STIP study, it was observed that the number of previous DMARDs did not affect the drug persistence [43]. Braña et al. noted that previous therapies with bDMARDs did not influence drug persistence, whereas the prior use of methotrexate, in contrast with our study, was linked to a higher ixekizumab discontinuation [44]. Weddell et al. reported additionally comparable drug survival rates in PsA patients treated with IL-17 inhibitors, regardless of whether they were bDMARD-naïve or had prior exposure to bDMARDs [49]. 

These characteristics were analyzed to create multivariate analyses aimed at identifying factors that might affect DRR. However, it seems that even when using these models, they do not appear to predict improved DRR.

In our cohort of patients characterized with multiple comorbidities, many of whom had experienced treatment failure with second-line therapies, ixekizumab furthermore exhibited a favorable safety profile. 

The discontinuation due to AEs occurred in 15.0% of patients, a higher percentage compared to the literature [58]. However, it is important to note that the AEs were not severe. Indeed, the most common AEs that resulted in the discontinuation of treatment were localized or diffuse skin reactions, which, however, were not severe and were in line with the expectations, as they had been already previously described in previous randomized controlled trials and real life data (SPIRIT-P1, SPIRIT-P2, SPIRIT-P3, and SPIRIT-H2H) [14,15,17,58,61,62].

Notably, our study revealed no severe infections. Similarly, Bastard et al. described serious infections in only 2.9% of the 344 patients treated with ixekizumab [52]. No severe infections were detected even among patients who were tested positive for SARS-CoV-2. Also, this finding aligns with the existing literature, which does not exhibit an increased risk of SARS-CoV-2-associated hospitalization or mortality in patients undergoing treatment with IL-17i [63]. Only one patient had to discontinue treatment due to an infectious disease, specifically persistent oral candidiasis. Chronic fungal diseases were also described in the study of Weddell et al. [49]. Localized candida infections were reported in a low proportion of patients treated with ixekizumab, likely related to the function of IL-17A in defense against these pathogens [44,62]. An analysis of administrative claims in the USA revealed that the risk of serious infection in patients treated with IL-17i appears to be similar to that in patients treated with TNFi [41].

Within our cohort, one patient exposed to ixekizumab developed microscopic colitis. Cases of microscopic colitis occurring during IL-17i therapy have already been documented in the worldwide pharmacovigilance database [64], and IL-17i were previously associated with increased rates of AEs and the exacerbation of symptoms related to Crohn’s disease [65,66]. A rate of new cases of Crohn’s disease and ulcerative colitis of less than 1% were documented in PsO patients [67]. As seen from a pooled analysis of the open-label period of SPIRIT-P1, SPIRIT-P2, and SPIRIT-P3, the rate of chronic inflammatory bowel diseases among PsA patients treated with ixekizumab was 0.2% [68]. Hence, although the evidence suggests a low rate of new onset of inflammatory bowel disease among patients exposed to ixekizumab, careful screening seems relevant especially in presence of symptoms suspicious for IBD. Considering real-life data, comparable findings were presented by Braña et al., where the predominant AEs were cutaneous and gastrointestinal, including a new diagnosis of Crohn’s disease [44].

Lastly, a patient received a diagnosis of lung cancer shortly after beginning therapy, despite screening performed prior to prescription with a chest X-ray. However, the diagnosis occurred soon after the induction period, so a direct link to ixekizumab administration seems unlikely.

Our study has certain limitations, primarily due to its retrospective monocentric design, which led to a lack of data concerning minor AEs, especially those that did not result in treatment discontinuation, as well as in the assessment of disease activity.

On the other hand, based on the current literature in the field, our cohort is among the largest and most well-characterized monocentric real-world cohorts of patients on ixekizumab so far, thereby offering valuable real-life insights on ixekizumab use in PsA patients and providing important data about DRR and safety, particularly among patients with multiple comorbidities.

In conclusion, in our study, ixekizumab revealed a good DRR and safety profile, even among patients with multiple treatment failures, comorbidities, and obesity, which are common in PsA patients. The prior use of methotrexate did not affect DRR in our study, in contrast to previous findings [44]. Additionally, we observed comparable efficacy in both peripheral and axial phenotypes, despite a negative trend in the latter. Moreover, our data suggest that initiating ixekizumab earlier may provide additional benefits. However, addressing these points will require larger sample sizes and further studies.

## Figures and Tables

**Table 1:** Demographic, clinical features, and discontinuation of therapy.

PsA Patients’ Characteristics and Treatment	80 Patients
Demographic characteristics	
Age, years, mean ± SD, years	50.1 ± 11.8
Male sex, n. (%)	24 (30.0)
BMI, mean ± SD, kg/m^2^	28.1 ± 5.4
Obesity (BMI ≥ 30 kg/m^2^), n. (%)	27 (33.8)
Smoking habit, n. (%)	22 (27.5)
Clinical characteristics	
Disease duration median (IQR), years	4 (9)
Disease duration ≤ 2 years, n. (%)	30 (37.5)
Disease duration ≥ 5 years, n. (%)	38 (47.5)
Disease duration ≥ 10 years, n. (%)	21 (26.3)
HLA-B27, n. (%)	5 (6.3)
Peripheral involvement, n. (%)	80 (100.0)
Skin involvement, n. (%)	68 (85.0)
Axial involvement, n. (%)	25 (31.3)
Enthesis involvement, n. (%)	35 (43.8)
Psoriatic onychopathy, n. (%)	19 (23.8)
Comorbidities	
Comorbidity, n. (%)	46 (57.5)
High blood pressure, n. (%)	36 (45.0)
Type 2 diabetes, n. (%)	17 (21.3)
Fatty liver disease, n. (%)	17 (21.3)
Cardiovascular disease, n. (%)	16 (20.0)
Dyslipidemia, n. (%)	12 (15.0)
Kidney disease, n. (%)	5 (6.3)
Previous treatment	
Previous therapy with NSAIDs n. (%)	39 (48.8)
Previous therapy with GCs n. (%)	45 (56.3)
Previous therapy with csDMARDs n. (%)	65 (81.3)
Previous therapy with MTX n. (%)	61 (76.3)
Previous therapy with bDMARDs n. (%)	49 (61.3)
Previous therapy with tsDMARDs n. (%)	6 (7.5)
Previous TNFi n. (%)	46 (57.5)
Previous IL-12/23i n. (%)	4 (5.0)
Previous IL-17i n. (%)	12 (15.0)
Previous tsDMARDs n. (%)	9 (11.3)
Failure to 1 bDMARD/tsDMARD n. (%)	32 (40.0)
Failure to 2 bDMARDs/tsDMARDs n. (%)	15 (18.8)
Failure to 3 or more bDMARDs/tsDMARDs n. (%)	9 (11.3)
Ixekizumab treatment	
Ongoing at the last observation n. (%)	43 (53.8)
Discontinuation due to inefficacy n. (%)	25 (31.3)
Discontinuation due to primary inefficacy n. (%)	9 (11.3)
Discontinuation due to secondary inefficacy n. (%)	16 (20.0)
Discontinuation due to AEs n. (%)	12 (15.0)

Footnotes: PsA, psoriatic arthritis; BMI, body mass index; SD, standard deviation; IQR, interquartile range; HLA, Human Leukocyte Antigen; NSAID, non-steroidal anti-inflammatory drugs; GCs, Glucocorticoids; csDMARDs, conventional synthetic Disease-Modifying Antirheumatic Drugs; MTX, methotrexate; bDMARDs, biologic Disease-Modifying Antirheumatic Drugs; tsDMARDs, targeted synthetic Disease-Modifying Antirheumatic Drugs; TNFi, tumor necrosis factor inhibitor; IL-12/23i, interleukin 12/23 inhibitor; IL-17i, interleukin 17 inhibitor; AEs, adverse events.

**Table 2:** Baseline clinical predictors of ixekizumab discontinuation through multivariate analysis.

Clinical Variables	OR	95%CI	*p* Value
Discontinuation of Ixekizumab			
Multivariate analysis			
Age	0.99	0.97–1.03	0.980
Male Sex	1.21	0.60–2.27	0.591
Disease duration ≤ 2 years	1.51	0.76–2.99	0.235
HLA-B27	2.72	0.90–8.23	0.076
Multivariate analysis			
Age	0.99	0.96–1.02	0.991
Male Sex	1.42	0.64–3.16	0.386
Comorbidity	1.10	0.47–2.57	0.825
Obesity	0.78	0.37–1.64	0.511
Multivariate analysis			
Age	1.01	0.98–1.03	0.922
Male Sex	1.09	0.53–2.25	0.811
Axial Disease	1.67	0.86–3.25	0.133
Skin involvement	0.89	0.38–2.07	0.791
Multivariate analysis			
Age	0.99	0.97–1.03	0.989
Male Sex	1.17	0.57–2.43	0.664
Previous csDMARDs	1.26	0.49–3.28	0.630
Previous bDMARDs	1.07	0.52–2.19	0.852

Footnotes: OR, odds ratio; CI, confidence interval; HLA, Human Leukocyte antigen; csDMARDs, conventional synthetic Disease Modifying Antirheumatic Drugs; bDMARDs, biologic Disease Modifying Antirheumatic Drugs.

## Data Availability

The original contributions presented in the study are included in the article/Supplementary Materials, and further inquiries can be directed to the corresponding author.

## Supplementary Materials

The following supporting information can be downloaded at: https://www.mdpi.com/article/10.3390/jpm14070716/s1. Table S1: Adverse events that led to discontinuation.
