# Supplementary material for: Retention Rate of Ixekizumab in Psoriatic Arthritis: A Real-World Study
Source: J Pers Med. 2024 Jul 3;14(7):716. doi: 10.3390/jpm14070716 (PMC11278385; doi:10.3390/jpm14070716)
Supplement: Supplementary file 1 [file jpm-14-00716-s001.zip › jpm-3066337-supplementary.pdf]

Journal of Personalized Medicine

MDPI

Supplementary Materials:

Supplementary Table S1. Adverse events that led to discontinuation.

	**80 patients**
Discontinuation due to AEs, n (%)	12 (15.0)
Local skin reaction, n (%)	4 (5.0)
Diffuse skin reaction, n (%)	3 (3.75)
Diarrhoea, n (%)	3 (3.75)
Lung malignancy, n (%)	1 (1.3)
Infective disease, n (%)	1 (1.3)

Footnotes: PsA, psoriatic arthritis; AEs, adverse events. One patient presenting diarrhea was thereafter diagnosed with microscopic colitis.
